# Recalcitrant Fungal Prosthetic Joint Infection of the Knee Cleared Using Voriconazole via Intra‐Articular Catheters

**DOI:** 10.1002/ccr3.71257

**Published:** 2025-10-14

**Authors:** Matthew Hnatow, Blake C. Martin, Emma Herrera

**Affiliations:** ^1^ Orthopaedic Center of Corpus Christi Corpus Christi Texas USA

**Keywords:** arthroplasty, fungal infection, intra‐articular, joint infection, prosthetic join infection

## Abstract

Prosthetic joint infection (PJI) is a high‐cost and high‐morbidity complication of total joint arthroplasty accounting for about 1%–2% of all joint replacements. Rarely, a fungal pathogen may cause coinfection or superinfection and complicate treatment. Fungal PJI is associated with higher morbidity and a lower rate of clearance of infection, with rates as low as 50% or less in the literature. Intra‐articular antibiotics as a treatment for PJI may be a promising intervention for challenging PJIs. We present a novel case utilizing voriconazole via intra‐articular catheters with successful clearance of a recalcitrant fungal prosthetic joint infection with 2 years' follow‐up. A detailed case study and protocol is presented for a 71‐year‐old male patient who failed two 2‐stage revision arthroplasties for complicated and recalcitrant fungal PJI. The patient was indicated for intra‐articular administration of antifungal medication. A two‐stage, both‐component knee revision with placement of two indwelling intra‐articular catheters was performed. Subsequently, voriconazole was administered daily for 6 weeks in alternating catheters. After completion of antifungal treatment, the catheters were removed in clinic. The patient demonstrates no evidence of infection after 2 years with a return to baseline function. To our knowledge, this is the first case in which intra‐articular voriconazole is administered through indwelling catheters of the knee. We demonstrate successful clearance of a complex and recalcitrant fungal PJI using this novel treatment. Intra‐articular use of voriconazole may be an option for patients with fungal PJI.

**Summary**

- The use of voriconazole via intravenous Hickman catheters may be used to effectively treat complex periprosthetic fungal infections following knee arthroplasty.

## 1 Introduction

Prosthetic joint infection (PJI) is a high‐cost and high‐morbidity complication of total joint arthroplasty accounting for about 1%–2% of all joint replacements [1]. PJI is more common in total knee arthroplasty (TKA) compared to total hip arthroplasty (THA) [1]. Furthermore, revision joint replacement has a higher probability of PJI compared to primary joint replacement [1]. Some preoperative risk factors include active infection, prior local infection, or prior local surgery [1]. Postoperative risk factors include immune suppression, inflammatory arthropathy, and lifestyle factors such as smoking, obesity, and alcohol [1]. PJI is typically due to Staphylococcus species, mainly *Staphylococcus aureus*, although fungal causes can occur with Candida species ( *Candida albicans* ) being the most common pathogen [1]. Rarely, these fungal pathogens may cause coinfection or superinfection and complicate treatment. Fungal PJI is associated with higher morbidity and a lower rate of clearance of infection with rates as low as 50% or less in the literature [2]. Intra‐articular antibiotics as a treatment for PJI may be a promising intervention for challenging fungal PJIs as well. We present a novel case utilizing voriconazole via intra‐articular catheters with successful clearance of a recalcitrant fungal prosthetic joint infection with 2 years' follow up. Voriconazole was used because amphotericin was on nationwide backorder at the time.

## 2 Case History/Examination

The patient was a 71‐year‐old man with a history of a left total knee arthroplasty with another surgeon. His postoperative course was complicated by prosthetic joint infection. He was taken for a staged revision with an articulating spacer with the index surgeon. During his antibiotic course, cultures initially remained negative, and he had recurrence of purulence while on broad‐spectrum antibiotics. Shortly after or around that point, he was taken for a repeat stage 1 with another articulating spacer with the index surgeon. There was difficulty clearing his infection once again. Subsequently, his cultures were positive for *Candida albicans* , and he was switched to an oral antifungal. At this point, he presented to us as a second opinion with recalcitrant fungal PJI. We offered staged revision, with tumor‐style debridement, a static spacer this time, and use of Hickman catheters for administration of intra‐articular antibiotics. Although there is always a risk of failure or recurrence, we feel these methods result in a high likelihood of success. The patient had failed all appropriate attempts for conservative management and desired surgical intervention. Our discussion included risks, benefits, and alternatives of surgery including expectations and rehabilitation. For this, risks include pain, bleeding, infection, damage to surrounding structures, failure of procedure, need for more procedures, deep vein thrombosis, pulmonary embolism, stiffness, numbness, and risks of anesthesia including death. Before surgery, we had him on an antibiotic holiday (no antibiotics to allow any latent infections to potentially manifest).

## 3 Differential Diagnoses, Investigation, and Treatment

**FIGURE 1:**
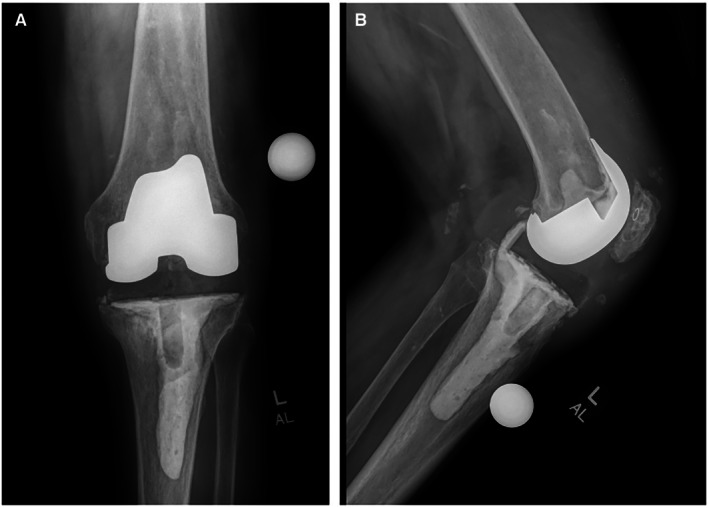
(A) Preoperative anteroposterior x‐ray of the knee. (B) Preoperative lateral x‐ray of the knee.

The patient had his primary knee arthroplasty in 2021, which was complicated by infection, and two 2‐stage revisions by the same surgeon. The initial cultures were negative. After the second revision, cultures then showed fungus, *Candida albicans* . At this time, the patient then presented to us for another opinion. His C‐reactive protein (CRP) was > 100 mg/L. There was no draining wound. His range of motion (ROM) was 45°–100°. He was on broad‐spectrum antibiotics with oral Diflucan. We discussed an antibiotic holiday and repeating the 2‐stage surgery, using the Stryker TS revision knee system, with the patient. The patient's surgery was then scheduled. The preoperative x‐rays of the patient's knee are shown in Figure 1A,B.

During stage 1 of the procedure, there was frank purulence throughout the knee, with the main pocket being posteromedially at the femur. A static spacer was placed using an intramedullary knee‐spanning carbon fiber rod. Three tissue specimens were sent to the lab for culture. Two Hickman catheters were placed intra‐articularly. The postoperative plan consisted of weight bearing through the leg, no range of motion, and a peripherally inserted central catheter (PICC) line.

The Hickman catheter protocol that was followed and successfully initiated with voriconazole was as follows. Administration of intra‐articular antibiotics via the Hickman catheter was performed under sterile conditions. Prior to each infusion, physician orders were reviewed and all necessary supplies (antibiotic syringe, chlorhexidine gluconate swabs, sterile luer lock cap, and povidone‐iodine swab) were prepared. Hand hygiene was performed and sterile gloves were applied. The infusion port was disinfected with chlorhexidine for 30 s and allowed to dry completely before connection. Using aseptic technique, the antibiotic syringe was attached, the catheter unclamped, and the antibiotic administered slowly. The catheter was then reclamped, and the syringe removed. The port was subsequently disinfected with povidone‐iodine and secured with a sterile luer lock cap to create a Betadine seal. Ports were alternated systematically, with port #1 used on odd‐numbered days and port #2 on even‐numbered days. If resistance was encountered during infusion, the alternate port was used, and the treating physician was notified if patency could not be established in either lumen. Notably, the catheter was not flushed with saline at any time. The insertion site was covered with sterile split gauze and Tegaderm, which were changed as needed until in‐growth occurred and drainage resolved. The clinical team was instructed to notify the physician promptly in the event of purulent drainage, erythema, or swelling at the insertion site, as well as in cases of catheter dislodgement, damage, or loss of patency.

**FIGURE 2:**
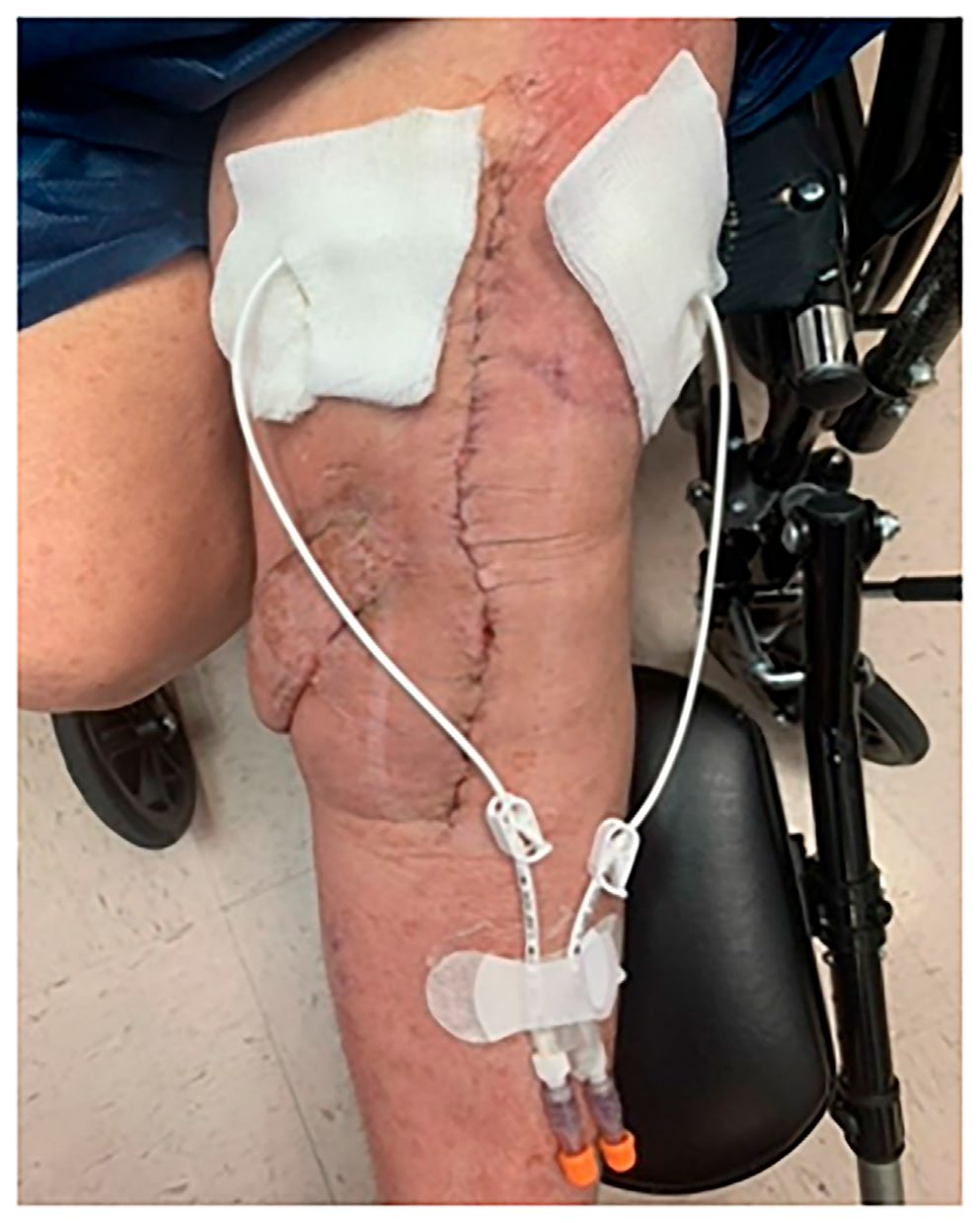
Hickman catheters applied to the patient.

Antibiotic infusion was once a day. The dose/volume administered is consistent, which differs from the original Hickman protocol with vancomycin, which starts small then increases gradually to achieve a maintenance dose. Weekly labs should include blood urea nitrogen (BUN), serum creatinine, and serum voriconazole level. The protocol dosages are 200 mg voriconazole/5 mL sterile water each day for 6 days. The Hickman catheters applied to the patient are shown in Figure 2.

At the first postoperative visit, the patient reported intermittent, burning‐type pain in the lateral and anterior aspects of the knee. The patient was ambulating with a rollator walker at this time. The wound VAC was removed, and no erythema or drainage was seen, although mild effusion was noted, indicating the wound was healing well. The Hickman catheter protocol was successfully initiated and was proceeding without complication. We then discussed wound care, medications, and planned follow‐up for one week. At the follow‐up, the patient had continued pain on the anterior aspect that was worse during ambulation, as well as global tenderness. The staples were removed, no drainage was seen, and mild effusion was evident. Tissue cultures from the time of stage 1 confirmed the presence of Candida species (*albicans*). Tramadol was prescribed, and the patient continued on IA antibiotics. Weight bearing as tolerated was instructed.

After 6 weeks of successful treatment, antimicrobial treatments were discontinued, and the PICC line and Hickman catheters were removed. Hickman catheter removal was done in clinic, at bedside, under local anesthesia. The most recent ESR was 15 mm/h, and CRP was 6 mg/L. We discussed clearance of infection from ESR and CRP trends, as well as the clinical picture, and proceeding to stage 2 re‐implantation. During stage 2 revision, there were healthy appearing tissues throughout, arthrofibrosis, and the knee was stable after revision. Tissues were then sent for culture (×3). Full weight bearing with routine follow‐up was planned, and oral prophylactic antibiotics and cefadroxil were prescribed. The cefadroxil was prescribed because some evidence had shown lower PJI rates in patients who used extended antibiotic prophylaxis [3]. At the postoperative visit, the patient had no fever, chills, wound redness, or draining, or any concerning symptoms. The cultures were negative from the time of surgery but were held for a fungal culture. On inspection, mild serosanguinous drainage was present and was removed with the wound VAC. The knee ROM was 5°–100° and was stable to stress tests. One week later, the patient had no complaints. The patient was undergoing physical therapy, and the staples were removed. On inspection, there was mild drainage from the knee, ROM was 5°–100°, and the knee was stable to stress tests. The cultures were negative at this time, and we discussed continued physical therapy (PT) and x‐rays on our next visit.

**FIGURE 3:**
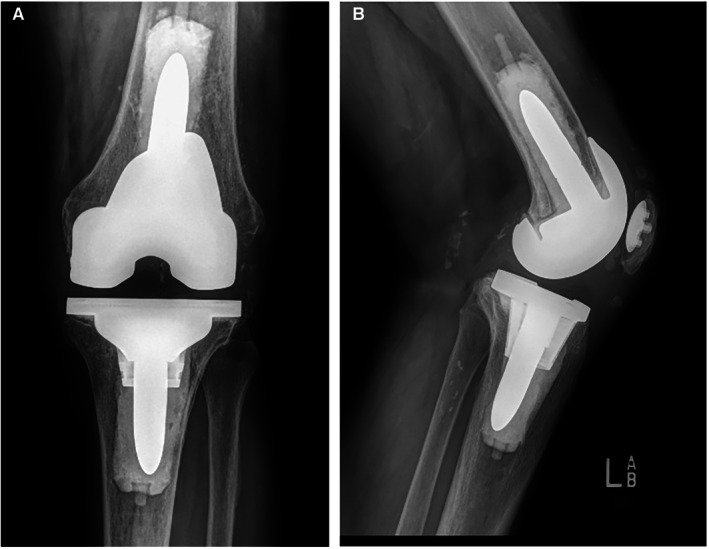
(A) Postoperative anteroposterior x‐ray of the knee. (B) Postoperative lateral x‐ray of the knee.

Three weeks later, the patient presented with serous drainage with yellow discoloration from his incision. There was a small open wound along the incision inferior to the midline of the knee joint. ROM was 0°–115°. X‐rays were obtained and there were no concerning findings. We planned to follow up in 1 week for a wound check. We suspended PT for wound rest. The postoperative x‐rays of the patient's knee are shown in Figure 3A,B.

At the follow‐up, the patient presented with persistent drainage. There appeared to be a localized suture reaction vs. wound dehiscence present. Due to continued drainage in this high‐risk setting, we discussed a washout procedure or long‐term antibiotics. We recommended incision and drainage (I&D) with evaluation of the arthrotomy and possible polyethylene exchange. We then scheduled the I&D. At the I&D, two small areas resembling a suture abscess with no clear tract through the arthrotomy and no frank purulence were noted. The arthrotomy was left intact, and cultures were taken (×3).

At the postoperative visit, the patient was reporting no concerning symptoms and was ambulating with the assistance of a walker. The bandage and wound VAC were removed. The incision was healing well with no open wounds or drainage. Cultures from the suture abscesses revealed Staph epidermitis. The patient was placed on oral antibiotics with Rifampin and Linezolid as a precaution, and the patient was ordered to resume PT. The Rifampin and Linezolid were decided to be prescribed after discussion with an infectious disease physician for *Staphylococcus epidermidis* sensitivity. One week later, the patient was ambulating with assistance, mild aches, and no concerning symptoms. Most staples were removed. There was some mild drainage, so two staples were left near the drainage. One week after that, the patient had minimal pain, no concerning symptoms, and mild drainage. The remaining staples were removed, and the patient continued antibiotics. Three weeks after that, the patient reported no pain, was ambulating with assistance, and antibiotics were completed. There was no drainage, the incision was healed well, and there were no open wounds.

## 4 Conclusion and Results

During the 6‐week follow‐up, the patient reported no signs of drainage and had mild occasional pain. The passive ROM was 0°–120°. The active ROM revealed a mild 10° lag. The knee was stable with stress tests. There were no open wounds and no effusion. The patient appeared completely clear of infection and superinfection. The patient was released with no restrictions until the 1 year follow‐up. At the 1‐year follow‐up, 2‐year follow‐up, and to the present date of manuscript submission, the patient is clear of infection.

## 5 Discussion

Intra‐articular catheters work by administering antibiotics directly into a joint. The use of intra‐articular catheters in PJIs has become more talked about in recent years, with Hickman catheters being the most frequent type of catheter used [4]. Bactericidal levels to treat planktonic infections are not hard to achieve in joint fluid with IV antibiotics, which is why we treat native septic arthritis with systemic antibiotics with excellent success rates [5, 6]. However, in PJI, systemic antibiotics fail because they can't achieve minimal biofilm eradication concentrations (MBEC), which can be much higher than the minimum inhibitory concentration (MIC) for planktonic bacteria [6, 7]. Biofilms are present on prosthetic materials and bone, which is why IA works, by achieving local concentrations above the MBEC, thereby helping to eradicate local biofilms [6, 7]. Bactericidal levels of antibiotics are difficult to achieve in infected total joint arthroplasty when intravenous antibiotics or antibiotic‐loaded cement spacers are used, but intra‐articular (IA) delivery of antibiotics has been effective in several studies [7]. Intra‐articular infusion of antibiotics may be a viable option for PJI treatment, as it has a theorized benefit of delivering a drug in high concentrations with more precise dosage control compared to other methods of administration (e.g., systemic, IV) [6]. Further advantages of intra‐articular administration include improved treatment success by maintaining sustained high intra‐articular antimicrobial concentration effective against biofilm pathogens, reduced systemic exposure to antimicrobials which minimizes systemic toxicity, and enhanced biofilm eradication [4, 8, 9]. Adverse effects of intra‐articular antibiotics include renal failure, catheter leakage or failure, and local inflammatory reactions that should resolve with discontinuation of catheters [8, 10]. Chondrotoxicity may also be possible when high concentrations of intra‐articular antimicrobials are used [10].

Most studies in current literature utilizing this approach (> 85%) used vancomycin and imipenem as the antibiotics of choice [4]. This is expected as most PJIs are bacterial in origin [1, 4]. However, a case series analyzed eleven case reports of intra‐articular treatment of fungal PJI, seven of which had satisfactory outcomes [11]. We found another study demonstrating the use of intra‐articular catheters on fungal (candida) PJIs [12]. This study was a case series made up of two patients that were successfully treated with no evidence of recurrence on follow‐up [12]. However, the antibiotic utilized in this study was amphotericin B (standard treatment), which differs from our study [12]. Another study utilized IA micafungin as the antibiotic of choice for a fungal PJI, which also differs from our study [13]. A later study utilized oral and IA voriconazole to treat a fungal PJI of the knee, while levofloxacin was also prescribed because bacterial infection couldn't be ruled out [14]. This is very similar to our study; however, we did not use oral voriconazole alongside the IA voriconazole as the previous study did [14].

In our study, voriconazole was used because amphotericin B was on nationwide backorder. Another study utilized the exact same approach, intra‐articular voriconazole for fungal infection, but the fungal pathogen was *Fusarium solani* [15]. Furthermore, the treatment was used to treat arthritis following bone marrow transplant, not PJI as seen in our case [15]. Our case report is unique because not only did we treat a fungal PJI ( *Candida albicans* ), but we also utilized voriconazole as the sole intra‐articular antibiotic. To our knowledge, no other studies have utilized this exact treatment method for a fungal PJI.

There are limitations to this report. This was a single patient treated with this treatment method, limiting the generalizability of the patient results. Furthermore, this was a sole case report with no other identical cases in current literature to compare and analyze with, further limiting the generalizability of the results. While the outcome was successful, there is no comparison to standard treatments such as amphotericin B or systemic antifungals alone. The use of Hickman catheters requires a diligent aseptic technique, which may not be feasible in all settings or for all patients. The protocol described could be adapted for similar cases, but it is essential to proceed with careful patient selection and monitoring.

To our knowledge, this is the first case in which intra‐articular voriconazole was administered through indwelling catheters of the knee to treat PJI. We demonstrate successful clearance of a complex and recalcitrant fungal PJI using this novel treatment. This case report shows that intra‐articular use of voriconazole may be an option for patients with fungal PJI. Further studies should be conducted to evaluate the effectiveness and patient outcomes following utilization of this treatment to further the validity and generalizability of this case report.

## Author Contributions

**Matthew Hnatow:** conceptualization, investigation, project administration, supervision, validation, visualization, writing – original draft, writing – review and editing. **Blake C. Martin:** conceptualization, investigation, methodology, visualization, writing – original draft, writing – review and editing. **Emma Herrera:** conceptualization, investigation, methodology, visualization, writing – original draft.

## Consent

The authors have obtained written informed consent from the patient.

## Conflicts of Interest

The authors declare no conflicts of interest.

## Data Availability

The data that support the findings of this study are available on request from the corresponding author. The data are not publicly available due to privacy or ethical restrictions.
